# Proteomics Profiling of Chikungunya-Infected *Aedes albopictus* C6/36 Cells Reveal Important Mosquito Cell Factors in Virus Replication

**DOI:** 10.1371/journal.pntd.0003544

**Published:** 2015-03-04

**Authors:** Regina Ching Hua Lee, Justin Jang Hann Chu

**Affiliations:** Laboratory of Molecular RNA Virology and Antiviral Strategies, Department of Microbiology, Yong Loo Lin School of Medicine, National University Health System, National University of Singapore, Singapore; Centers for Disease Control and Prevention, UNITED STATES

## Abstract

Chikungunya virus (CHIKV) is the only causative agent of CHIKV fever with persistent arthralgia, and in some cases may lead to neurological complications which can be highly fatal, therefore it poses severe health issues in many parts of the world. CHIKV transmission can be mediated via the *Aedes albopictus* mosquito; however, very little is currently known about the involvement of mosquito cellular factors during CHIKV-infection within the mosquito cells. Unravelling the neglected aspects of mosquito proteome changes in CHIKV-infected mosquito cells may increase our understanding on the differences in the host factors between arthropod and mammalian cells for successful replication of CHIKV. In this study, the CHIKV-infected C6/36 cells with differential cellular proteins expression were profiled using two-dimensional gel electrophoresis (2DE) coupled with the use of matrix-assisted laser desorption/ionization time-of-flight mass spectrometry (MALDI-TOF MS). 2DE analysis on CHIKV-infected C6/36 cells has shown 23 mosquito cellular proteins that are differentially regulated, and which are involved diverse biological pathways, such as protein folding and metabolic processes. Among those identified mosquito proteins, spermatogenesis-associated factor, enolase phosphatase e-1 and chaperonin-60kD have been found to regulate CHIKV infection. Furthermore, siRNA-mediated gene knockdown of these proteins has demonstrated the biological importance of these host proteins that mediate CHIKV infection. These findings have provided an insight to the importance of mosquito host factors in the replication of CHIKV, thus providing a potential channel for developing novel antiviral strategies against CHIKV transmission.

## Author Summary

Chikungunya virus (CHIKV), a re-emerging mosquito-borne virus, is the main cause of CHIKV fever, persistent arthralgia and serious neurological complications which can be highly fatal; therefore, it poses serious health threats worldwide. Unraveling the underappreciated aspects of mosquito cellular factors that contribute to the replication processes of CHIKV was performed using two-dimensional gel electrophoresis (2DE) coupled with the use of matrix-assisted laser desorption/ionization time-of-flight mass spectrometry (MALDI-TOF MS). The 2DE proteomics profiling of CHIKV-infected mosquito C6/36 cells has revealed twenty-three proteins that were differentially regulated upon CHIKV infection. These proteins are shown to be involved in diverse biological pathways and cellular processes. Notably, upon selected genes knockdown, spermatogenesis-associated factor, enolase phosphatase e-1 and chaperonin-60kD are found to be important during the replication processes of CHIKV. This study illustrates the importance of mosquito cellular factors in association with CHIKV infection in mosquito cells and reveals an interesting portal for developing novel antiviral strategies in CHIKV studies.

## Introduction

Chikungunya virus (CHIKV) is an emerging mosquito-borne virus of the genus Alphaviruses, family *Togaviridae*. The long-neglected viral disease was first discovered in Tanzania in 1952. CHIKV is an enveloped, single-stranded, positive-sense RNA virus with a genome size of approximately 12,000 nucleotides. The viral genome encodes for five different structural proteins which include the capsid (C) and E1, E2, 6K and E3 as well as four non-structural proteins (nsP1–P4). Two of the structural proteins (E1 and E2), are embedded in the lipid bilayer surrounding the viral nucleocapsid [1,2]. Both E1 and E2 proteins are able to facilitate the early entry processes for CHIKV, by directing the virus particles to the host cells for attachment and fusion with cellular membranes upon entry into cells [3–5].

CHIKV transmission to human occurs by *Aedes* (*Ae*.) species mosquitoes, mainly *Ae*. *aegypti* and *Ae*. *albopictus*. However, in the CHIKV epidemic in 2005–06 on La Réunion Island, the main vector that was responsible for transmission between humans was the Asian tiger mosquitoes, *Ae*. *albopictus*. This species is well-known to be susceptible to CHIKV infection. Notably, this mosquito species is also responsible for high morbidity rates which accounted for millions of serious yet non-fatal cases [6]. Additionally, the mutation on the E1 structural envelope glycoprotein of the CHIKV particle (Ala-226-Val) was noted to be responsible for epidemic progression. This mutation has resulted in the change of the primary transmission vector from *Ae*. *aegypti* to *Ae*. *albopictus* [6].

Alphaviruses can be broadly classified as New World encephalitic viruses or Old World arthitogenic viruses. CHIKV is categorized together with other broadly recognized Old World alphaviruses such as Sindbis (SINV), Semliki Forest (SFV) and Ross River (RRV) viruses [7,8]. Chikungunya has been described as “bent walker”, where hunched posture was often observed in patients suffering from persistent arthralgia. Chikungunya disease is often characterized by polyarthralgia, high fever, joints pain and sometimes a maculopapular rash. Development of the symptoms after being bitten by a CHIKV-infected mosquito vector is usually around 4–7 days. [9]. Acute CHIKV infection only lasts about 1–10 days. On the other hand, chronic CHIKV infection often persists for longer periods [3,5].

Previous proteomics studies have been conducted to understand the importance of human host factors in neuronal and hepatocytes-like cell lines upon CHIKV-infection [10–13]. These studies have revealed the essential roles of host proteins in association with CHIKV replication, either by facilitating the replication process or inhibiting the production of infectious CHIKV virions. Apart from the usual CHIKV-infection symptoms, recent CHIKV-infected patients were also found to suffer from myositis [3–5]. This CHIKV outbreak in La Réunion Island has been reported with a high percentage of myositis cases, leading to a recent proteomics profiling study of CHIKV infection of the human rhabdomyosarcoma cells, SJCRH30. Investigation of CHIKV-infection in a mammalian cell line has shown new evidence of host cellular proteins mechanisms involved in viral replication and progression of disease in human [14]. In addition, proteome analysis studies were also conducted on mosquito vectors revealing dynamic differences in cellular proteins involved in virus replication when compared with mammalian cells. In particular, the well-studied vector, *Ae*. *aegypti*, revealed that upon CHIKV infection in salivary glands, cytoskeleton proteins such as beta-tubulin was found to be down-regulated, while up-regulation of beta-tubulin protein was observed in mammalian cell, SJCRH30 upon CHIKV-infection [14,15]. However, other cellular proteins such as protein disulfide isomerase (PDI), a multifunctional protein that plays an important role in regulation of redox reactions on cell surfaces behaved otherwise upon CHIKV-infection [16,17]. Proteome analysis studies showed that upon CHIKV-infection in both mammalian cells and salivary glands of *Ae*. *aegypti*, PDI was observed to be down-regulated at the later time-point of CHIKV-infection [14,15].

However, little is known about the proteome of CHIKV-infected *Ae*. *albopictus* mosquito cells. Therefore, this study aims to reveal the proteome of CHIKV-infected *Ae*. *albopictus* C6/36 mosquito cells by the means of advance proteomics profiling platform technologies including two-dimensional gel electrophoresis (2DE) coupled with the use of matrix-assisted laser desorption/ionization time-of-flight mass spectrometry (MALDI-TOF MS). The proteomics analysis results of CHIKV-infected *Ae*. *albopictus* C6/36 cells has revealed various mosquito proteins, which are involved in different biological processes such protein processing, phagosome trafficking, oxidative phosphorylation, metabolic mechanisms, lysosome function, ubiquitin-mediated proteolysis, RNA degradation, phototransduction and cysteine and methionine metabolism. Moreover, results from siRNA knockdown of chaperonin-60kD, enolase phosphatase e-1 and spermatogenesis-associated factor genes suggested the important roles of these cellular proteins during the replication of CHIKV within the infected *Ae*. *albopictus* mosquito cells.

## Materials and Methods

### Cell culture

*Ae*. *albopictus* C6/36 cells (ATCC, CRL-1660) and baby hamster kidney (BHK-21) cells (ATCC, CCL-10) were used in this study. C6/36 cells were cultured in Leibovitz-15 (L-15) growth medium (Sigma-Aldrich Corp., St Louis, MO, USA), which contained a 10% heat-inactivated fetal bovine serum (FBS) (Hyclone, Cramlinton, UK) at 28°C in the absence of CO_2_. BHK-21 cells were cultured in Rosewell Park Memorial Institute (RMPI) 1640 growth medium (Sigma-Aldrich Corp), which contained a 10% fetal bovine serum (FBS) (Hyclone) at 37°C in the presence of 5% CO_2_.

### Viruses and CHIKV infection

CHIKV strain SGEHICHD122508 (Accession No.: FJ445502.2) kindly provided by Environmental Health Institute (EHI), National Environment Agency (Singapore). Low passage CHIKV stock (Passage number = 3) was used throughout this study to mimic the virus infection as close as to the environment to prevent laboratory adaptation of virus on cell lines. CHIKV was propagated in a T75 flask of C6/36 cells and incubated for 1.5 hours at 37°C in 5% CO_2_. After which, the virus was supplied with L-15 medium which contained 2% heat-inactivated FBS and maintained in 28°C in the absence of CO_2_. Virus was harvested at 4 days post-infection (d.p.i.). The virus titre was determined using viral plaque assay on BHK-21 cells and expressed as plaque-forming units (PFU/mL).

### CHIKV infection of C6/36 cells

*Ae*. *albopictus* C6/36 cells (5×10^6^ cells) were seeded into T-25 cell culture flasks. The cells were then infected with CHIKV-122508 at multiplicity of infection (M.O.I.) 10 and incubated at 37°C in the presence of 5% CO_2_ for 1.5 hours. The excess virus was washed off with 1X Phosphate Buffer Saline (PBS) and L-15 medium supplemented with 2% heat-inactivated FBS was added. After 24, 48 and 96 hours post-infection (h.p.i.), C6/36 cells were washed with 1 mL wash buffer thrice, provided from ProteoExtract Complete Mammalian Proteome Extraction Kit (Millipore) A cell scraper was used to scrap the monolayer of cells into the 1 mL of wash buffer. The cell pellet was obtained by centrifugation at 150×g for 10 minutes at 4°C and stored at -80°C for further analysis.

### Cellular protein extraction

Cellular protein extraction of C6/36 cells was carried out with the use of the ProteoExtract Complete Mammalian Proteome Extraction Kit (Millipore) according to the instructions provided by the manufacturer. The cell pellets of different time points of CHIKV-infection (24, 48 and 96 h.p.i) were resuspended in an ice cold resuspension buffer. The cell pellet was then lysed using room temperature extraction reagent provided. Reducing agent and enzyme (Benzonase Nuclease) were added into the suspension to disrupt disulfide bonds and allow degradation of RNA and DNA. The fully solubilized suspension was centrifuged for 30 minutes at 16,000×g at 4°C and stored at -20°C. RC DC Bradford assay (BioRad) was used to determine the protein sample concentrations with BSA (0.5mg/mL) as the standard solution.

### Two dimension gel electrophoresis (2DE) and image analysis

250 μg of protein sample was loaded into individual lanes on the focusing tray. The pre-wetted electrode wicks were overlaid with 11cm (pH 3–10) ReadyStrip IPG Strip (BioRad). The IPG strips were subjected to passive rehydration for 12 hours, before exposing to isoelectric focusing on Protean IEF Cell i11 (BioRad). The following conditions used in the 2DE run were: 250V for 20 minutes with linear ramp, 8,000V for 2.5 hours with linear ramp and 8,000V for 25,000 v-hours with rapid ramping. After isoelectric focusing, the IPG strips were equilibrated with pre-warmed DTT Equilibration Buffer I (BioRad) followed by iodoacetamide-Equilibration Buffer II (BioRad), for 10 minutes each on orbital shaker. The equilibrated IPG strips were further resolved by 12.5% Tris-HCl Criterion gel (11cm) (BioRad) at 200V for 65 minutes. After resolving the IPG strips, the gels were stained with InstantBlue (Expedeon) for 1 hour. Destaining was done thrice with water to remove excess stains on the gel. The gels were then scanned using GS-800 Calibrated Densitometer (BioRad). Detection of spots, spots matching and spot intensity analysis were performed using PDQuest 2-D Analysis Software (BioRad). Two biological repeats were obtained from two independent CHIKV-infection experiments.

### Spots selection using PDquest 2-D analysis software (BioRad)

Protein spots selection was performed by comparison of mock-infected vs CHIKV-infected gels at 24 h.p.i. A 2-fold changes was selected as the threshold value. Unwanted spots that were not within the 2-fold threshold value were filtered off and the remaining spots were of interest to this study. Based on the protein spots selected from the first time point of 24 h.p.i, further analysis was carried out on the same 23 protein spots for their differential regulation in the later time points of 48 h.p.i and 96 h.p.i. The threshold value of 2-fold change does not apply to these later time points as the selected hits were predetermined at 24 h.p.i.

### In-gel digestion and MALDI-TOF MS

Each excised protein spots was sent for in-gel digestion and MALDI-TOF MS was kindly carried out by Protein and Proteomics Centre, National University of Singapore (Singapore). A MASCOT program (http://www.matrixscience.com) was used to identify the proteins by searching in the National Centre for Biotechnology Information non-redundant (NCBInr) database. There was no thresholding algorithm applied to the MS/MS fragment ion intensities.

### Bioinformatics

The functional and localization studies of the selected protein spots were performed using KEGG Pathways (http://www.genome.jp/kegg/pathway.html) and database from Swiss-Prot/TrEMBL (http://www.uniprot.org/uniprot). The protein-protein interactions between the protein spot were analyzed using STRING network v9.05 (http://string-db.org).

### Small interfering RNA (siRNA) reverse transfection

A set of siRNAs targeting the different *Ae*. *albopictus* genes were selected to perform siRNA reverse transfection assays in C6/36 cells, including Chaperonin-60kD, ch60 partial mRNA (NCBI Accession: XM_001661714.1), Enolase phosphatase e-1 partial mRNA (NCBI Accession: XM_001657643.1) and Spermatogenesis associated factor partial mRNA (NCBI Accession: XM_001654630.1) and were purchased from Sigma-Aldrich Corp., St Louis, MO, USA. The siRNAs were diluted from 100 μM stock concentration to working concentrations of 0.1, 1.0, 10, 30 and 50 nM with DharmaFECT Cell Culture Reagent (DCCR) (Thermo Scientific) and transfection reagent (Dharmafect-1) to make up a final volume of 100μL per well. After which, siRNAs were incubated for 30 minutes at room temperature. The positive control was replaced with DCCR. 1.2×10^6^ C6/36 cells in 400 μL were seeded into 24-well plate together with the siRNA-transfection mixture. After 48 hours post-transfection, the siRNA-transfected cells were subjected to CHIKV-infection with a M.O.I. 10 for 24 h.p.i. The viral supernatant was harvested and viral plaque assays were performed.

### Cellular cytotoxicity assay

C6/36 cells (2.4×10^5^ cells) were seeded on 96-well plates and the cells were transfected with siRNA for 48 hours post transfection. After which, the cells were washed twice with 1X Phosphate Buffer Saline (PBS) before addition of alamarBlue reagent (Invitrogen) supplemented L-15 medium containing 2% heat-inactivated FBS. The cells were then incubated at 37°C in the presence of 5% CO_2_ for 3 hours. With the use of the microplate reader (Infinite 200, Tecan), the fluorescence signals were captured using at the excitation wavelength at 570 nm and emission wavelength at 585 nm.

### RNA quantification via qRT-PCR

Gene expression validation was performed by qRT-PCR. Total RNA was extracted from C6/36 cells with RNeasy Extraction Kit (Qiagen) after gene silencing. The samples were then assayed in a 25 μl reaction mixture containing 12.5 μl SYBR Green Master Mix (Roche, Switzerland), 1 μl forward and reverse primer respectively, 25 ng of RNA, 1 μl reverse transcriptase and 8.5 μl nuclease free water. A non-template control was also included. The cycling conditions for one-step SYBR Green-based RT-PCRs consisted of a 30-min reverse transcription step at 42°C and 10 minutes of Taq polymerase activation at 95°C, followed by 40 cycles of PCR with denaturation occurring at 95°C for 15 seconds, with annealing and extension taking place at 60°C for 30 seconds. Following amplification, a melting curve analysis was performed at 70–95°C to verify the melting temperature of PCR products amplified by the *Ae*. *albopictus* gene primer pairs. The primers pairs used in this experiments are: Chaperonin-60kD (Forward, 5′-CTGCCGTCGAGGAAGGTATC-3′, Reverse, 3′- CTGGTCTTCCGGCCTTAACT-5′), Enolase phosphatase e-1 (Forward, 5′-CAGTCTGTAGAAAGCCATCG-3′, Reverse, 3′-GCATTCCTACCTTTATTTGATTGG-5′) and Spermatogenesis-associated factor (Forward, 5′-AGTTGGAAGAGTCATCGTTT-3′, Reverse, 3′-GCTACCTATACCTGCTCCTACTA-5′) and actin controls (Forward, 5′-CCACCATGTACCCAGGAATC-3′, Reverse, 3′-CACCGATCCAGACGGAGTAT-5′).

### Statistical analysis

Statistical analyses were performed with the use of one-tailed Student’s t-test. The significance level was determined at *p* < 0.05 (*), *p* < 0.01 (**) or *p* < 0.0001 (***). Data obtained throughout the study were from three independent experiments.

## Results

### Profiles of CHIKV-infected C6/36 cells using two dimensional gel electrophoresis (2DE)

**Fig 1:**
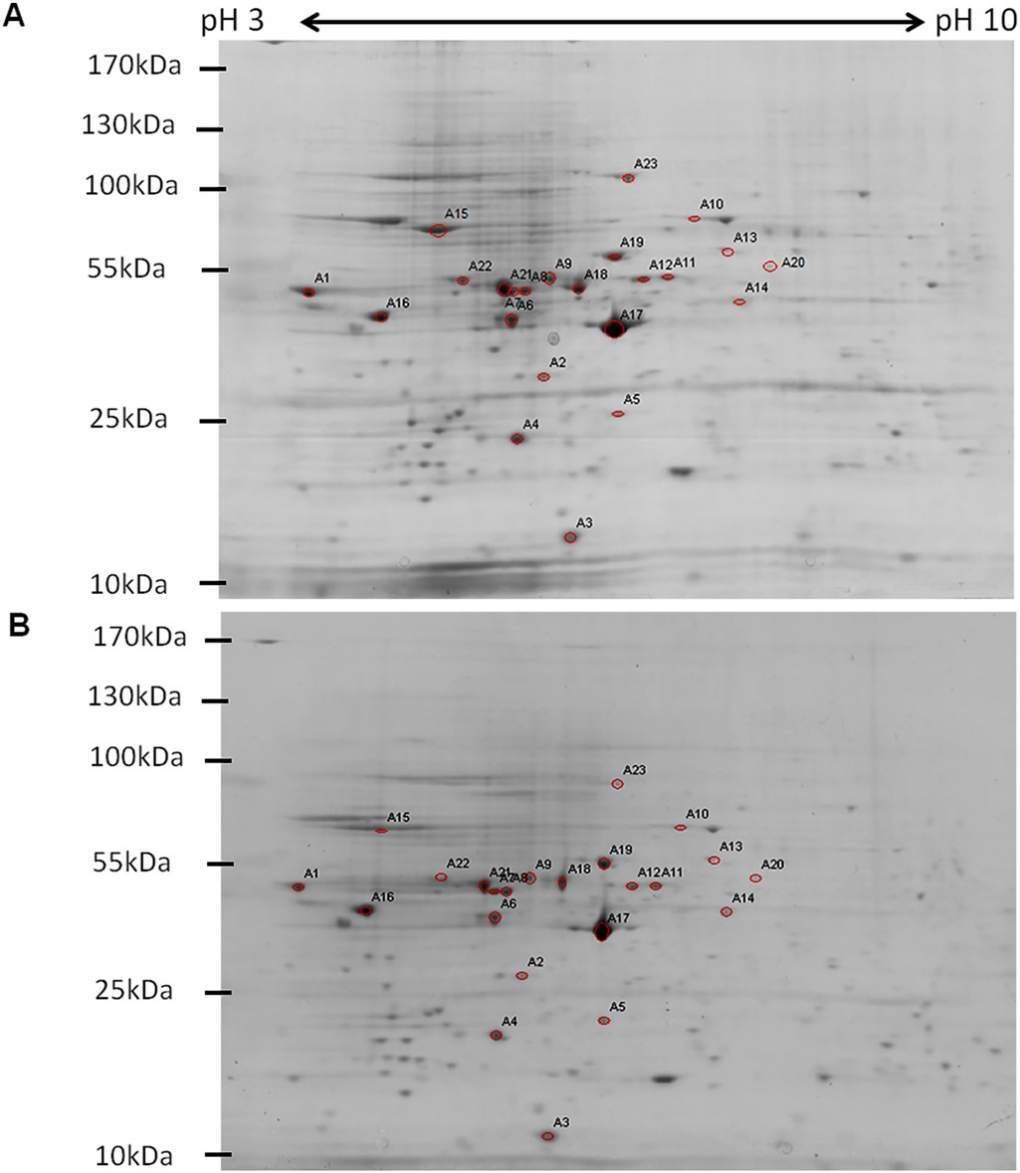
2DE analysis of CHIKV-infected C6/36 cells. (A) Twenty-three protein spots of mock-infected samples that showed 2-folds changes with *p*<0.05 (Student’s *t*-test) as compared to (B) CHIKV-infected samples were annotated. All the SDS-PAGE gels from the second dimension separation were analyzed using PDQuest 2-D Analysis Software (BioRad). Two biological replicates of the CHIKV-infected samples from each time-point infection were grouped and compared against to the corresponding biological replicates of the mock-infected samples. Representative gels are from 24h mock-infected and 24h CHIKV-infected lysates.

To elucidate the expression of the different host cellular proteins in response of CHIKV-infection in C6/36 cells, the total cell lysates of CHIKV-infected and mock-infected C6/36 cells were extracted with the use of the ProteoExtract Complete Mammalian Proteome Extraction Kit from various time-points post-infection (24, 48 and 96 h.p.i.). The efficiency of the extraction kit was equally efficient in the extraction of the complete insect cell proteome performed on C6/36 cells when compared to mammalian cell lines. High protein concentrations were obtained when determined with the RC DC Bradford assay (BioRad). 2DE analysis of samples was performed in a total protein concentration of 250 μg/gel. Two independent experiments were performed and the protein samples were harvested in two biological repeats (data not shown). Approximately 50 to 80 spots were detected across different time-points of infection. At 24h and 48h time-points, a large number of protein spots were detected in both mock-infected and CHIKV-infected C6/36 samples. However, at 96h time-point of infection, fewer protein spots were observed on the protein gel. Fewer spots detected might be attributed to cell death induced by CHIKV infection as infection progressed. A total number of twenty-three protein spots showed at least 2-fold dynamic changes with *p* < 0.05 (Student’s *t*-test) when comparison were made between the mock-infected and CHIKV-infected samples at 24 h.p.i as shown in Figs. 1A and 1B. The different protein spots were then excised and sent for MALDI-TOF MS/MS analysis. The results of the MALDI—TOF MS/MS were searched against the NCBInr database for protein identification using MASCOT program (http://www.matrixscience.com).

**Fig 2:**
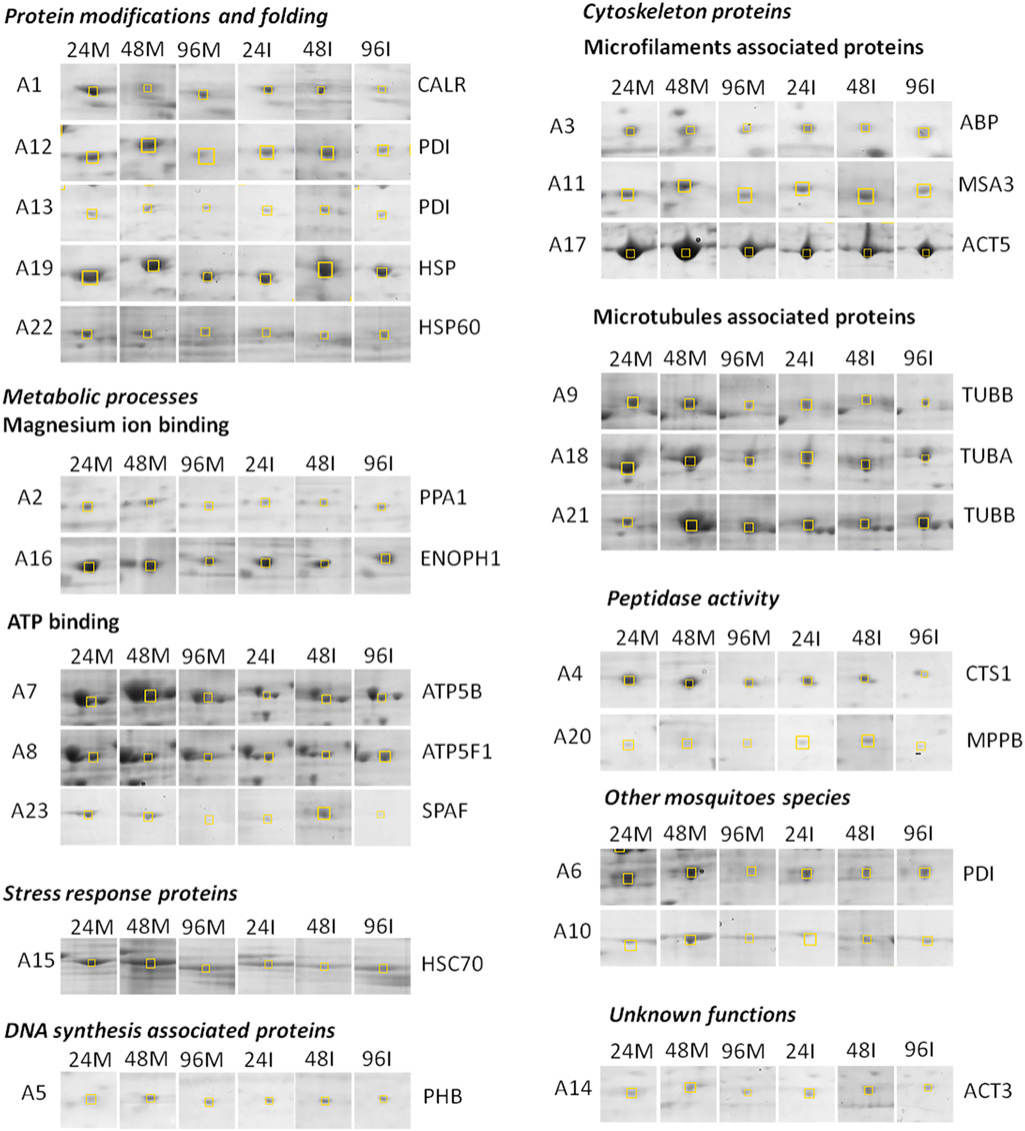
2-DE dynamic profiles of the selected host proteins upon CHIKV-infection at M.O.I. 10. Yellow box indicates the differentially expressed proteins. Mock-infection for each time-point served as a control, as shown in panel 24M, 48M and 96M. CHIKV-infection profiles analyzed at 24, 48 and 96 h.p.i., as shown in the panel of 24I, 48I and 96I, respectively. The functional grouping of each protein is shown in UniprotKB database.

Expression profiles of the 2DE dynamic protein at different time-points of CHIKV-infection are shown in Fig. 2. As shown in Table 1, upon CHIKV-infection in C6/36 cells, 14 proteins were up-regulated at 24 h.p.i., 13 proteins were up-regulated at 48 h.p.i., and 12 proteins were up-regulated at 96 h.p.i., respectively. Furthermore, 9 proteins showed down-regulation at 24 h.p.i., while 11 proteins spots were down-regulated at 48 h.p.i and 11 proteins showed down-regulation at 96 h.p.i, respectively (Table 1).

**Table 1:** Mean change in spot intensities and mass spectrometry data of 23 differentially regulated proteins from CHIKV-infected C6/36 cells at M.O.I. 10.

Spot	Mean Change (%)			Protein name	Mascot score	Peptides identified	Sequence coverage (%)	Mr (Dalton)	Calculated pI	NCBI ID
	24 hpi	48 hpi	96 hpi							
***Protein modifications and folding***										
**A1**	-9	2047	-22	Calreticulin	399	20	44	46983	4.42	gi|157113061
**A12**	-4	16	-11	Protein disulfide isomerase	293	11	20	55211	4.95	gi|157118649
**A13**	173	9	-3	Chaperonin	569	25	49	53596	5.59	gi|157116575
**A19**	285	511	384	Chaperonin-60kD, ch60	809	29	48	61155	5.47	gi|157129785
**A22**	29	98	12	Protein disulfide isomerase	209	17	28	56260	4.95	gi|157107430
***Metabolic process***										
**Magnesium ion binding**										
**A2**	-1	-46	39	Inorganic pyrophosphatase	191	9	12	42275	6.17	gi|157135065
**A16**	67	5	4	Enolase phosphatase e-1	302	21	16	120591	4.34	gi|157112950
**ATP binding**										
**A7**	3290	-25	2991	ATP synthase beta subunit	787	26	39	53940	5.02	gi|157136033
**A8**	28	-9	-27	F0F1 ATP synthase beta subunit	751	21	30	53937	5.03	gi|94468834
**A23**	86	20	19	Spermatogenesis associated factor	77	21	33	80289	5.44	gi|157132226
***Cytoskeleton proteins***										
**Microfilaments associated proteins**										
**A3**	-9	5796	2573	Actin binding protein, putative	129	3	20	14697	5.49	gi|157136879
**A11**	-12	34	-13	Muscle-specific actin 3	208	11	30	41649	5.44	gi|33642245
**A17**	6	-15	47	Actin 5	709	25	62	42194	5.3	gi|67782283
**Microtubules associated proteins**										
**A9**	-1	-14	70	Tubulin beta chain	580	23	28	48748	4.79	gi|157132378
**A18**	-1	-3	-9	Tubulin alpha chain	975	31	63	50561	5.01	gi|157113931
**A21**	154	42	25	Tubulin beta chain	570	31	61	50543	4.75	gi|157132376
***Stress response proteins***										
**A15**	47	8	-23	Heat shock Cognate 70	1030	37	58	71388	5.31	gi|94468966
***Peptidase activity***										
**A4**	27	-40	-32	Cathepsin 1	163	6	13	38023	5.74	gi|157132324
**A20**	114	13	-55	Mitochondrial processing peptidase beta subunit	94	4	12	52843	5.87	gi|157109957
***DNA synthesis associated proteins***										
**A5**	**39**	-12	8	Prohibitin	373	14	33	29885	5.36	gi|157122974
***Other species***										
**A6**	-26	520	3050	Disulfide isomerase tigA	469	17	23	44733	4.91	gi|170052875
**A10**	279	1021	758	Conserved hypothetical protein	327	19	22	72845	5.91	gi|170036376
***Unknown functions***										
**A14**	666	1256	818	Actin A3	484	20	47	41865	5.47	gi|5751

Out of the 23 selected protein spots, 3 protein spots were identified from other species with unknown functions. The functional grouping of the proteins is based on the most significant function of each protein shown in Uniprot protein knowledge base, UniprotKB database (Swiss-Prot/TrEMBL; http://www.uniprot.org/uniprot).

### Mass spectrometric identification of differentially expressed proteins of C6/36 cells

Twenty-three protein spots were sent for MALDI-TOF MS/MS identification. Results showed that only 20 positive hits of protein spots showed differential up- or down-regulation from the *Ae*. *aegypti* protein database as the *Ae*. *albopictus* genome has not been sequenced. Three protein spots (Spot A6, A10 and A14) were shown to be from other mosquitoes species and functions were not identified (Table 1). Two different proteins out of the 20 positive protein hits from database were detected in more than one spot, namely protein disulfide isomerase (Spots A12 and A22) and tubulin-beta chain (Spots A9 and A21). They were detected with different molecular weight (MW) and isoelectric points (pI). This could be due to the different post-alternative splicing or post-translational modification (PTM) variants of the same protein.

**Fig 3:**
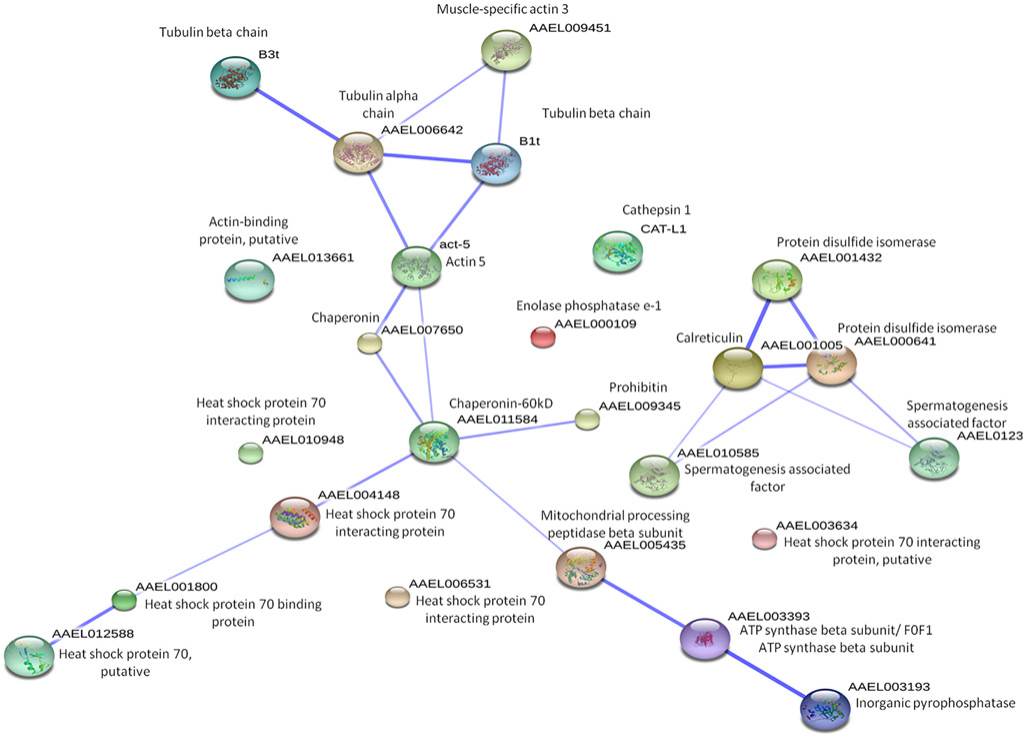
STRING analysis of protein-protein interactions between differentially expressed proteins identified through 2DE. The protein network analysis was performed using STRING v9.05. The interactions between expressed proteins are indicated by the connecting line. The thickness of the connecting line represents the strength of the associations. The interactions network is significantly enriched (*p*< 0.05).

**Fig 4:**
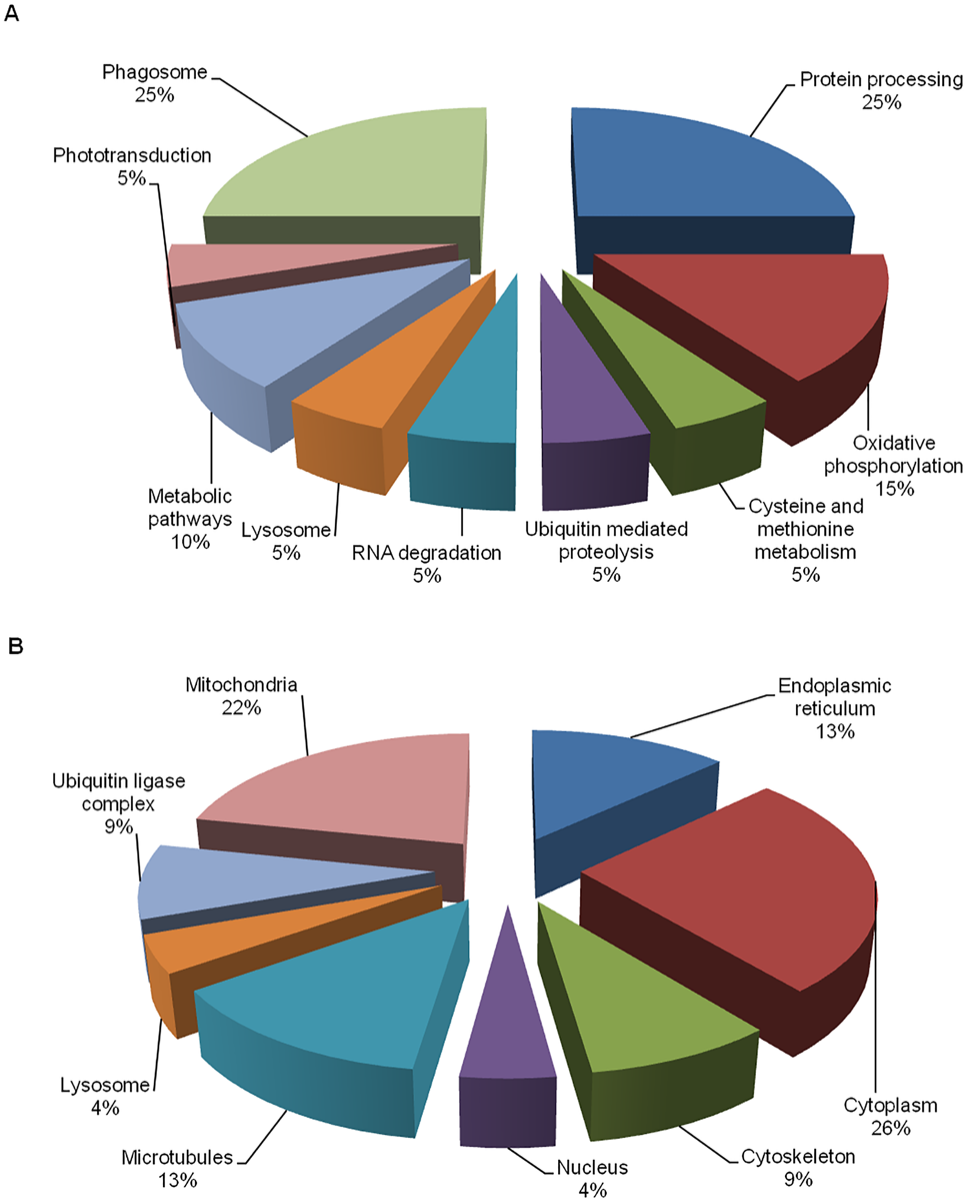
Cellular localization and functions of the differentially expressed proteins identified through 2-DE dynamic profiling. (A) The cellular localizations were determined using UniprotKB database. Most of the identified proteins were found in cytoplasm, and they mainly participate in protein processing. (B) The functions of each protein were determined using KEGG pathways database.

Additionally, STRING network analysis was used to analyze the protein-protein interactions of the identified proteins. STRING network database consists of the global resources such as well-studied and predicted protein interactions which come from different sources such as microarray analysis data, high-throughput studies, genomic context, and published literatures. This database will aid in the understanding of different biological processes carried out by the identified proteins that were being affected upon CHIKV-infection [18]. Proteins-protein interactions were shown in confidence view, whereby, twenty-five proteins interactions were observed in the STRING network analysis (Fig. 3). Although, twenty-three proteins were identified from mass spectrometry, a particular protein named HSP70 was detected in three other isoforms from STRING analysis because the mass spectrometry results did not indicate which HSP70 isoform was present. Therefore, they were included into the STRING analysis to allow the network to be more enriched. Furthermore, they are isoforms of HSP70 proteins, so their functions are similar to each other. The thickness of the connecting line of each protein where interactions of different proteins are observed in clusters indicates the strength of the interactions. These interacting proteins include the cytoskeleton proteins, protein modification proteins, metabolic process proteins and stress response proteins. They are connected together due to their similar functions. Protein names and their symbols are derived from UniprotKB and summarized in Fig. 3. In addition, based on the annotations from KEGG pathways database, the functional activities of these proteins are revealed. These identified proteins were mainly involved in protein processing and phagosome trafficking (25%), oxidative phosphorylation (15%), metabolic mechanisms (10%), lysosomal function, ubiquitin-mediated proteolysis, RNA degradation, phototransduction and cysteine and methionine metabolism (5%), respectively (Fig. 4A). Furthermore, the differential regulated proteins were categorized into different sub-cellular compartments and functions. The majority of the proteins are localized in the cytoplasm (30%), followed by microtubules and mitochondria (Fig. 4B).

As shown in Table 2, nine KEGG pathways were found to be significant where the *p*<0.05, was derived from false discovery rate (FDR) correction where errors are minimized when having multiple comparisons. This includes protein processing, phagosome trafficking, oxidative phosphorylation, metabolic mechanisms, lysosomal function, ubiquitin-mediated proteolysis, RNA degradation, phototransduction and cysteine and methionine metabolism.

**Table 2:** Nine significant KEGG pathways identified in STRING network.

KEGG Pathways	*p*-value
Phagosome	*p* < 0.001 (***)
Protein processing in endoplasmic reticulum	*p* < 0.05 (**)
Oxidative phosphorylation	*p* ≤ 0 (*)
Cysteine and methionine metabolism	*p* ≤ 0 (*)
Ubiquitin mediated proteolysis	*p* ≤ 0 (*)
RNA degradation	*p* ≤ 0 (*)
Lysosome	*p* ≤ 0 (*)
Metabolic pathways	*p* ≤ 0 (*)
Phototransduction	*p* ≤ 0 (*)

The pathways processes are determined using KEGG database. P-value was derived from the false discovery rate (FDR) correction to minimize errors from multiple comparisons.

### siRNA reverse transfection knockdown of targeted cellular genes in C6/36 cells

**Fig 5:**
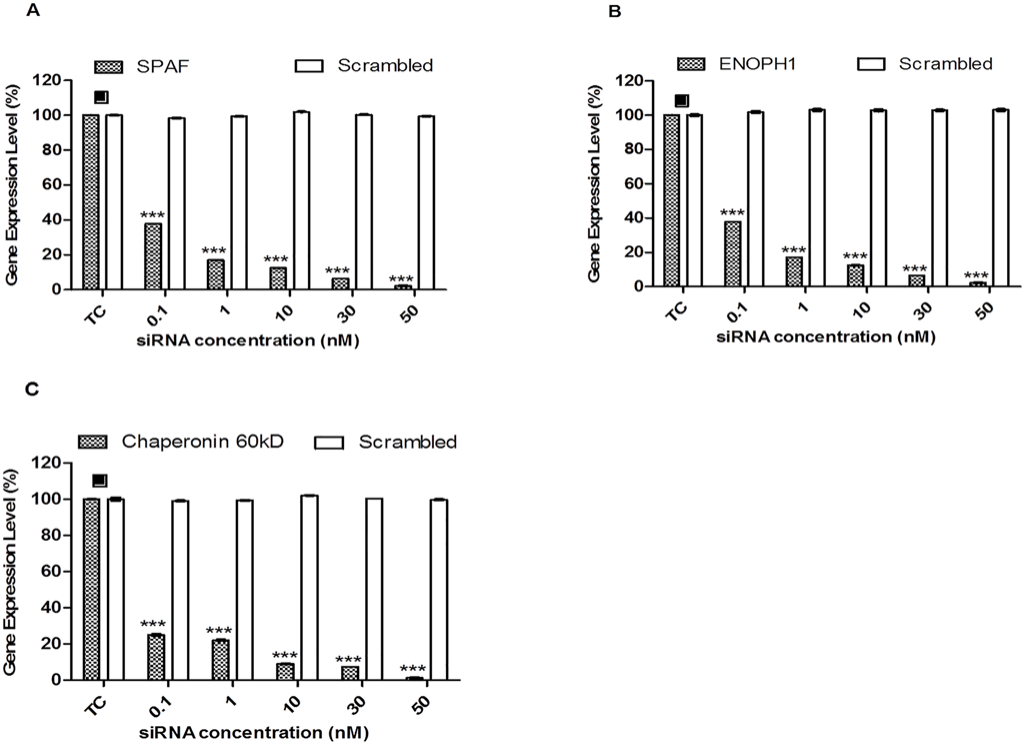
RNA levels on selected cellular genes. Scrambled siRNA-transfected cells (represented by white bars) against (A) Spermatogenesis-associated factor, (B) Enolase phosphatase e-1 and (C) Chaperonin-60kD show no knockdown of gene expression when compared to transfection control (TC). Cells transfected with targeted cellular siRNAs (represented by shaded bars) against (A) Spermatogenesis-associated factor, (B) Enolase phosphatase e-1 and (C) Chaperonin-60kD showed significant knockdown across all genes tested compared to transfection control (TC). The *asterisk* indicates *p values <0.05, **p values of <0.01 and ***p values <0.0001 by Student’s *t* test. Asterisks indicate statistically significant results relative to control group (■).

**Fig 6:**
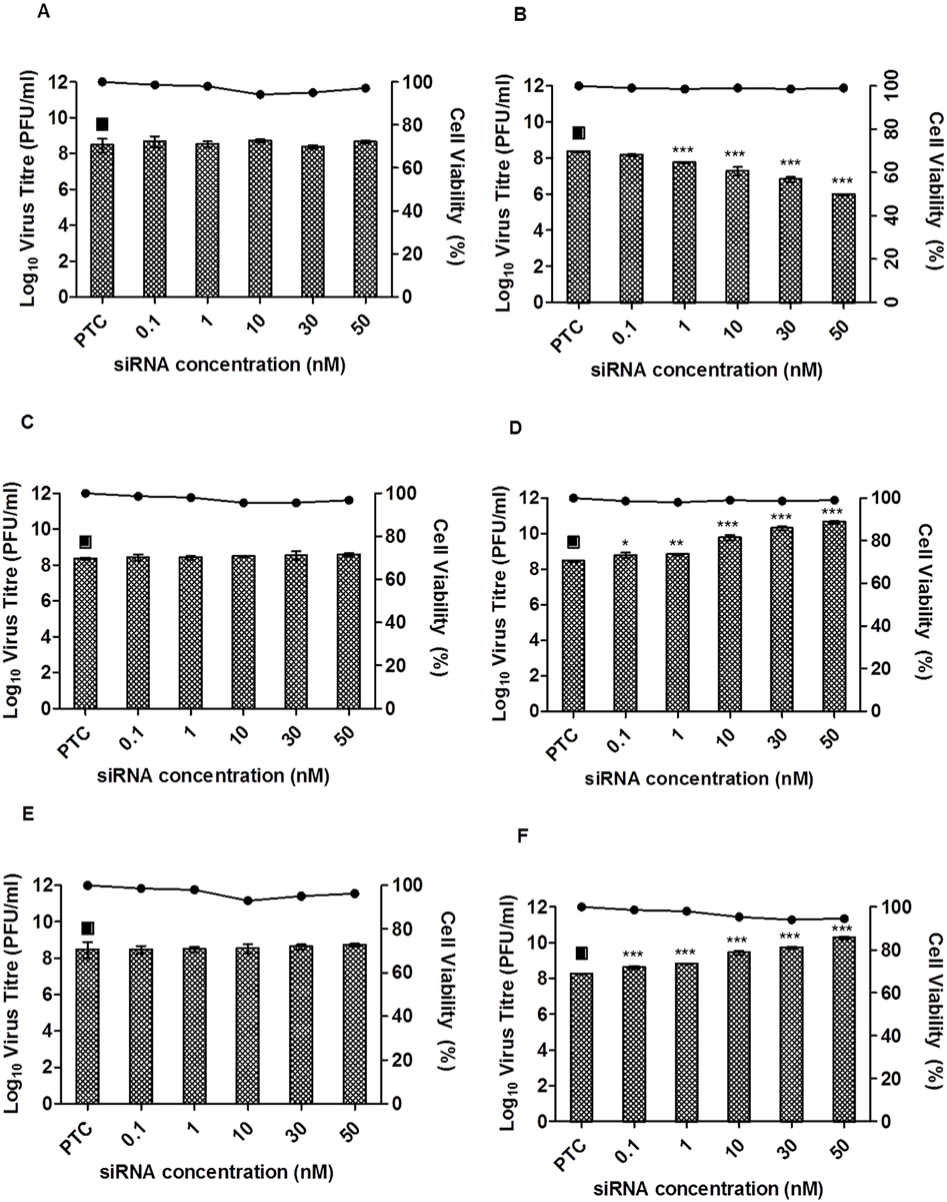
siRNA dose-dependent knockdown of Spermatogenesis-associated factor, Enolase phosphatase e-1 and Chaperonin-60kD. Scrambled siRNAs for (A) Spermatogenesis-associated factor, (C) Enolase phosphatase e-1 and (E) Chaperonin-60kD were subjected to transfection across a range of concentrations (0–50 nM) and then infected with CHIKV. No virus inhibition was observed for any scrambled siRNAs when compared to transfection control (TC). Gene-specific siRNAs against (B) Spermatogenesis-associated factor, (D) Enolase phosphatase e-1 and (F) Chaperonin-60kD were transfected into C6/36 cells at different concentrations (0–50 nM) and subjected to CHIKV infection. Significant dose-dependent inhibition of CHIKV infection was observed in spermatogenesis-associated factor from 30 nM to 50 nM, with approximately 2-log unit PFU/ml reductions. Significant dose-dependent increases in CHIKV titers were observed with enolase phosphatase e-1 and chaperonin-60kD from 10 nM to 50 nM, with approximately 2-log unit PFU/ml increases. Cell viability upon drug treatment is represented by the line graphs. The asterisk indicates *p values <0.05, **p values of <0.01 and ***p values <0.0001 by Student’s *t* test. Asterisks indicate statistically significant results relative to control group (■).

Data from the mass spectrometric results and the functional profiling shown by KEGG database and UniprotKB have revealed the differentially up and down regulated proteins that are involved in CHIKV infection. Therefore, to study the functional roles of these mosquito proteins related to CHIKV infection, three proteins, namely Spermatogenesis-associated factor, Enolase phosphatase e-1 and Chaperonin-60kD were selected for further downstream studies (Table 3). To investigate their roles in CHIKV infection, siRNA-mediated knockdown of these selected mosquito cellular genes was performed on C6/36 cells. To confirm efficacy of siRNA-mediated knockdown, the RNA expression levels of specific genes were evaluated by real-time PCR. Gene expression levels were observed to be significantly reduced for these three selected genes when compared with the control (TC) (Fig. 5). In contrast, there is minimal knock-down of the spermatogenesis-associated factor, enolase phosphatase e-1 and chaperonin-60kD with their, respective scrambled siRNA as shown in Figs. 6A, 6C and 6E, respectively.

**Table 3:** Small interfering (siRNA) sequences used in siRNA based experiments.

Small interfering (siRNA) sequences used in siRNA based experiments.		
Gene name	NCBI Accession number	Sequences
Aedes aegypti chaperonin-60kD, ch60 partial mRNA	XM_001661714.1	GCGAUUCCAAGCAUGUUGA, UCAACAUGCUUGGAAUCGC
Aedes aegypti enolase phosphatase e-1 partial mRNA	XM_001657643.1	GGAAGAAUCCGUAACAAGU, ACUUGUUACGAUUCUUCC
Aedes aegypti spermatogenesis associated factor partial mRNA	XM_001654630.1	GAUAUUCGCAAGUACGAAA, UUUCGUACUUGCGAAUAUC

Small interfering (siRNA) scrambled sequences used in siRNA based experiments.		
**Gene name**	**NCBI Accession number**	**Sequences**
Aedes aegypti chaperonin-60kD, ch60 partial mRNA	XM_001661714.1	AGGCGAUUCCAAGCAUGUU, AACAUGCUUGGAAUCGCCU
Aedes aegypti enolase phosphatase e-1 partial mRNA	XM_001657643.1	AAGAAUCCGUAACAAGUGG, CCACUUGUUACGGAUUCUU
Aedes aegypti spermatogenesis associated factor partial mRNA	XM_001654630.1	AUAUUCGCAAGAGUACGAA, UUCGUACUCUUGCGAAUAU

Spermatogenesis-associated factor (SPAF) belonging to a subfamily of the AAA-protein (ATPase Associated with diverse cellular Activities) has been reported to be involved in membrane fusion events [19–22]; it may play a role in CHIKV entry processes including endocytosis and fusion of CHIKV envelope with endosomal membrane for viral genome release into the cytoplasm of the infected cells. siRNA-mediated knockdown of spermatogenesis-associated factor (SPAF) gene was performed and the cells were subjected to CHIKV infection. A dose-dependent reduction in CHIKV virus titre was observed with a 1-log unit PFU/ml reduction at 30 nM, relative to the transfection control (PTC) samples (Fig. 6B). This result indicates the essential role of SPAF in the replication process of CHIKV within the mosquito cells.

Enolase phopsphatase e-1 (ENOPH1) is known to be a bifunctional enzyme that is responsible for the formation of acireductone 1,2-dihydroxy-3-keto-5-methylthiopentene [23] and is essential for the methionine salvage pathway, a process which converts methylthioadenosine (MTA) back into L-methionine [24]. Earlier study has shown that the methionine salvage pathway played an important role in stress responses by increasing the levels of polyamine, a protein essential for the growth and function of normal cells [25]. Under the same protein family, alpha-enolase—a metalloenzyme is able to cause specific humoral response in tumor cells [26]. Therefore, in this study, knockdown of ENOPH1 gene was carried out and interestingly the data revealed a 1.5 to 2 log unit PFU/ml increased in virus titre from 10 nM onwards when compared to PTC (Fig. 6D). Similarly, the knockdown of the chaperonin-60kD,—a chaperonin under the heat shock protein family, displayed a 2 log unit PFU/ml increase in virus titre upon knock down with increasing siRNA concentrations when compared to PTC (Fig. 6F). Chaperonin is an oligomeric protein that assists in proper folding of proteins in the cell in either normal or stress conditions [27–29]. It was also reported in previous studies that heat shock proteins are responsible as regulators in immune response [30,31]. In addition, to ensure that the effect on the virus titre upon siRNA-mediated knockdown was not cause by the siRNAs cytotoxicity, alamarBlue cytotoxicity assay was carried out (Fig. 6, line graph). Thus, these results may suggest that ENOPH1 and chaperonin-60kD may serve functional roles with important implications in regulating stress response and innate immunity of mosquito cells during CHIKV infection.

## Discussion

Proteomic approaches have been progressively gaining momentum over the years with the development of different proteome profiling methods such as SILAC, 2DE, tandem affinity purification (TAP) tagging and yeast-two hybrid screening. They have been actively used to investigate the host cellular responses upon virus infection [32]. Differential expression of host proteins during virus infection will provide us with insights to the importance of biological processes and cellular regulatory proteins affecting virus infection, replication and their release for transmission. In addition, the differences in protein expression between mammalian and arthropod hosts may enhance the current understanding on factors affecting transmission and epidemic progression. Recently, several studies evaluated the proteomic profiles of a variety of human and mosquito cell lines and mosquito vectors upon CHIKV-infection [10,12,14,33,34]. The *in-vivo* proteomics profile of the well-studied vector, *Ae*. *aegypti*, has been elucidated using two-dimension gel electrophoresis, while very few studies have been done on the recently emerged vector, *Ae*. *albopictus* [33,35]. Notably, the differential regulation of proteins in CHIKV-infected mosquito C6/36 cells is still unknown. In this study, we investigated proteomic profile of CHIKV-infected *Ae*. *albopictus* C6/36 cells using two-dimension gel electrophoresis. The proteome of CHIKV-infected C6/36 cells revealed many host proteins that were expressed differentially at the different time points of infection (24, 48 and 96 h.p.i). In particular, enolase phosphatase e-1, chaperonin-60kD and spermatogenesis-associated factor were found to be up-regulated across all time points of infected cells. A recent study on differentially regulated proteins in *Ae*. *aegypti* midguts reported an up-regulation of enolase and chaperonin 60-kD proteins upon DENV-2 infection but not in CHIKV infection [33]. This disparity may be attributed to the inherent differences in the mosquito vector used in the study since the cell line used in our investigation was derived from *Ae*. *albopictus*. Indeed, the proteomic profile of a CHIKV-infected cell lines are known to differ depending on the species [10,12,14].

A number of the differentially regulated proteins identified in this study are known to be involved in the host cellular stress pathways due to CHIKV infection (Table 1). In response to cellular stress, within the eukaryotic systems, a large number of highly conserved stress proteins were found to function as molecular chaperones and were up-regulated upon CHIKV infection. As mentioned in previous studies, these proteins are necessary for protein complexes folding, assembly and trafficking within cells. The production of these stress proteins can be caused by various external stimuli such as nutrients depletion and viral infection [36]. It is known that chaperonin binds to nascent polypeptide chains and partially folded intermediates of proteins, preventing protein aggregation and misfolding [28,37]. Garry and colleagues (1983) noted the involvement of stress proteins during early event of *Sindbis* infection and these events could be triggered by association of the viral capsid proteins with chaperonins [38]. Early literature revealed that the up-regulation of chaperone proteins in response to stress was induced by viral infection and was found in the endoplasmic reticulum (ER) where misfolded and unfolded proteins will trigger ER stress responses [39]. The infection of *Ae*. *albopictus* C6/36 cells by dengue virus similarly revealed that chaperone proteins up-regulation in the infected cells could be triggered by the stress response in the endoplasmic reticulum (ER) induced by viral infection [40]. Furthermore, chaperone proteins found in *Ae*. *albopictus* cells were also shown to interfere with Mayaro virus assembly and replication and protects the host cells during viral infection [41]. Notably, numerous *in vivo* and *in vitro studies* have revealed the relationship of chaperonin or heat shock proteins in association with the stimulation of innate and adaptive immune responses [42–44].

Chaperonin-60kD was noted to be up-regulated across the different time points in this study with the infection of C6/36 cells with CHIKV as shown in Table 1. Interestingly, the data from the siRNA-mediated knockdown of Chaperonin-60kD gene in CHIKV-infected mosquito cells showed an increase in virus titre, thus, our result may potentially suggest that Chaperonin-60kD may play a role in regulating the antiviral immune response against CHIKV infection (Fig. 6F).

Up-regulation of ENOPH1 has been shown in *Ae*. *albopictus* cells upon dengue infection and was postulated to be involved in the glycolysis pathway [40]. A previous study on influenza virus also displayed up-regulation of glycolytic enzymes in virus-infected cells indicating that upon virus infection, glucose metabolism in host cells was disturbed. It was mentioned by Ritter and colleagues (2010) that the increase in glycolysis activity was caused by the mitochondrial membrane breakdown during influenza virus infection, which decreased ATP concentration. Thus, activation of the glycolysis pathway was initiated to replace the lack of energy [45]. Previous literature has also demonstrated that alpha-enolase in the same enolase family is overexpressed upon hepatitis C virus (HCV) infection [46]. Furthermore, it was also mentioned that alpha-enolase is important in mediating both innate and acquired immunity [23,42]. The data from this study with the siRNA-mediated knockdown of ENOPH1 gene in C6/36 cells displayed an increased CHIKV titre as well as the up-regulation of ENOPH1 protein during CHIKV infection suggesting that ENOPH1 mediates CHIKV infection of mosquito cells when energy production is required (Table 1). At the same time, ENOPH1 may also exhibit a role in antiviral immune response against CHIKV infection in the mosquito cells (Fig. 6D).

The SPAF has been identified as a novel member in of the AAA-protein family (ATPase Associated with diverse cellular Activities) [19]. They function in a range of cellular processes, including membrane fusion [47,48]. A wide group of AAA-protein members have been shown to have important functions linked to membrane fusion events in different cellular organelles [19]. The first AAA-protein linked to membrane fusion is SEC18, an N-ethylmaleimide-sensitive fusion protein, that is necessary for secretory protein transportation between the ER and Golgi complexes [49]. Furthermore, two other types of AAA-proteins (CDC48 and p97ATPase) were also reported to be associated with membrane fusion events [47,48]. In our study, the up-regulation of SPAF protein during CHIKV infection was observed across all the time-points. It is possible that SPAF protein may be involved in membrane fusion events during the replication process of CHIKV within the infected mosquito cells. Previous studies have also shown that entry of alphaviruses lowers the pH within the endosomal vesicles and is needed for the fusion of the virus within the endosomal membrane of host cells for the release of the viral genome for replication [20,22]. Alternatively, SPAF protein may be required for the secretory protein transport of viral proteins for assembly and maturation. As shown previously by Chen and colleagues (2013) the exocytic vesicles containing mature CHIKV will fuse with the plasma membrane to release the virus via exocytosis. [45] Hence, upon siRNA-mediated knockdown of the SPAF gene, it showed a significant decrease in virus titre in a dose-dependent manner demonstrating the importance of SPAF in CHIKV infection (Fig. 6B).

In this study, chaperonin-60kD, ENOPH1 and SPAF displayed important functional roles during CHIKV infection in *Ae*. *albopictus* C6/36 cells. Essentially, our study has established that these set of proteins are associated with CHIKV infection and are essential for CHIKV replication. Therefore, elucidation of CHIKV infection into mosquito C6/36 cells will provide new insights of CHIKV viral pathogenesis and novel avenues for antiviral strategies.
